# The difference an end-of-life diagnosis makes: qualitative interviews with providers of community health care for frail older people

**DOI:** 10.3399/bjgp20X712805

**Published:** 2020-09-22

**Authors:** Louisa Polak, Sarah Hopkins, Stephen Barclay, Sarah Hoare

**Affiliations:** Palliative and End of Life Care Group;; Primary Care Unit, Cambridge Institute of Public Health, University of Cambridge School of Clinical Medicine, Cambridge Biomedical Campus, Forvie Site, Cambridge.; Primary Care Unit, Cambridge Institute of Public Health, University of Cambridge School of Clinical Medicine, Cambridge Biomedical Campus, Forvie Site, Cambridge.; Primary Care Unit, Cambridge Institute of Public Health, University of Cambridge School of Clinical Medicine, Cambridge Biomedical Campus, Forvie Site, Cambridge.

**Keywords:** terminal care, palliative care, community health services, frail elderly, aged

## Abstract

**Background:**

Increasing numbers of people die of the frailty and multimorbidity associated with old age, often without receiving an end-of-life diagnosis. Compared to those with a single life-limiting condition such as cancer, frail older people are less likely to access adequate community care. To address this inequality, guidance for professional providers of community health care encourages them to make end-of-life diagnoses more often in such people. These diagnoses centre on prognosis, making them difficult to establish given the inherent unpredictability of age-related decline. This difficulty makes it important to ask how care provision is affected by not having an end-of-life diagnosis.

**Aim:**

To explore the role of an end-of-life diagnosis in shaping the provision of health care outside acute hospitals.

**Design and setting:**

Qualitative interviews with 19 healthcare providers from community-based settings, including nursing homes and out-of-hours services.

**Method:**

Semi-structured interviews (nine individual, three small group) were conducted. Data were analysed thematically and using constant comparison.

**Results:**

In the participants’ accounts, it was unusual and problematic to consider frail older people as candidates for end-of-life diagnosis. Participants talked of this diagnosis as being useful to them as care providers, helping them prioritise caring for people diagnosed as ‘end-of-life’ and enabling them to offer additional services. This prioritisation and additional help was identified as excluding people who die without an end-of-life diagnosis.

**Conclusion:**

End-of-life diagnosis is a first-class ticket to community care; people who die without such a diagnosis are potentially disadvantaged as regards care provision. Recognising this inequity should help policymakers and practitioners to mitigate it.

## INTRODUCTION

The project of end-of-life care, like its parent project palliative care, evolved in the context of care for people with cancer^1^ before broadening to include all those with a life-threatening illness.^2^ In English end-of-life care policy, for example, a key commitment is to ensure good care for *‘every person nearing the end of their life’*.^3^ This commitment implicitly extends the remit of palliative care to encompass the growing proportion of deaths that occur in older people,^4^ many of whom die with frailty^5^ or multiple age-related comorbidities^6^ rather than with a single life-limiting condition. It is widely acknowledged, however, that providing good end-of-life care for this patient group offers challenges that may contribute to a *‘gap in the service’* ^7^ that has been identified as a key element of *‘disadvantaged dying’*.^7^^–^^9^

Policymakers place a growing emphasis on facilitating end-of-life care in community settings.^10^ Identifying candidates for such care is a central feature of policies and guidelines. This identification process, and the end-of-life ‘label’ it produces, functions like other kinds of clinical diagnosis, helping clinicians to identify treatment options, predicting outcomes and more broadly enabling access to services and status.^11^ The increased prominence of community end-of-life care and the central role of end-of-life diagnosis in recommendations are both illustrated by the recently revised GP contract for English primary care:^12^ establishing a palliative care register is incentivised, and *‘identifying patients in need of end-of-life care’* is described as the first *‘key* [step] *in the provision of high quality care at the end of life’*. The research literature, too, calls for GPs to extend and improve their palliative care^13^^,^^14^ and to emphasise the importance of earlier and more widespread diagnosis. This is an emphasis reflected in the Gold Standard Framework guidance^15^ to providers of community end-of-life care, and in the Royal College of General Practitioner’s Daffodil Standards.^16^

Following this end-of-life diagnosis-centred guidance is more challenging in people with multimorbidity and frailty than it is in people with cancer or an organ failure, because of their very different trajectories of health deterioration.^17^ While any end-of-life diagnosis has some inherent uncertainty until death, this uncertainty is particularly great in people who experience a gradual age-related decline in health over the last few months of life.^9^^,^^18^^,^^19^ So it is unsurprising that such people are more likely to die without an end-of-life diagnosis than, for instance, people with cancer.^14^ This study sought to establish how much this difference matters. To explore the ways in which having (or not having) an end-of-life diagnosis shapes access to care, the study drew on community providers’ accounts of caring for frail older people towards the end of their lives.

**Table:** How this fits in

It is well established that people who die of age-related multimorbidity are less likely to access adequate community end-of-life care than people who die of single conditions such as cancer. Current guidance encourages GPs to diagnose more frail older people as being close to the end of life, but the literature suggests that this is challenging, partly due to prognostic uncertainty. This study shows that community care providers prioritise people with an end-of-life diagnosis and offer them additional services, thus disadvantaging those who die without such a diagnosis. To help these potentially disadvantaged patients, clinicians and commissioners should consider basing decisions about allocating and prioritising care less on people’s prognosis and diagnosis, and more on their needs.

## METHOD

The participants were healthcare staff involved in the provision of community-based end-of-life care. They worked in a variety of contexts including out-of-hours (OOH) community nursing services, as well as institutions such as nursing homes and community hospitals, all within a single clinical commissioning group in England. All names have been changed in the study; each quotation in the text is followed by the participant’s place of work and a pseudonym. Table 1 provides a summary of participant roles.

**Table 1.** Study participants’ roles

**Interviewee role**	**Interviewees, *n***
*Individual interviews*	
Hospice matron	1
Coordinator for community end-of-life care	1
Community nurse manager (OOH)	3
Nursing home manager	2
Community hospital manager	2

*Group interviews*	
Group 1: Hospice doctors and managers	4
Group 2: Hospice nurses	2
Group 3: OOH/CCG doctors and managers	4

CCG = clinical commissioning group. OOH = out-of-hours.

One author conducted interviews in 2018 with 11 individuals and three small groups; the groups were formed in response to expediency and participants’ preferences. Interviews were semi-structured. Each occurred at the participant’s workplace and lasted approximately 45 minutes; all were audiorecorded and professionally transcribed. They were asked about their experiences of providing end-of-life care to frail older people. The interviewer avoided seeking clarification of terms like ‘frail’, ‘dementia’, or ‘old’, instead focusing on the way these categories were being used within participants’ accounts.

Interview data were analysed using thematic analysis with constant comparison.^20^ As group members’ interactions or prior relationships were not used as a source of analytic purchase, these interviews were not focus groups.^21^ An iterative analytic approach enabled a focus on ‘end-of-life diagnosis’, which was identified as a salient theme in the preliminary coding of the data.

## RESULTS

These data speak to two related questions concerning end-of-life diagnosis. First, they help characterise the kinds of people likely to receive an end-of-life diagnosis. Second, they demonstrate the power of such a diagnosis to shape care provision.

### Who gets an end-of-life diagnosis, and who does not?

Establishing an end-of-life diagnosis centres on estimating prognosis. In addition to the inherent uncertainty of these processes, there is wide variation in the way participants define the end of life:

*‘End of life, are we looking at,* [Hospice Charity 2] *often talk of end of life as the last week, seven days, but if you go to the Gold Standard Framework it’s the last year. So I would identify as the last year, is that okay? ’*

(Fiona, end-of-life community facilitator)

‘Elderly, frail at end of life, that could be anything from a few weeks up to 18–24 months.’

(Hattie, community nurse, OOH)

*‘These are end-of-life care beds* [...] *so we’re looking to take people in the last two to three weeks of their life.’*

(Bev, hospice)

Varied as these definitions are, they clearly play a big part in determining who gets an end-of-life diagnosis. But before choosing a definition and attempting to fit it to an individual, clinicians have to think about that individual as the right kind of person to consider for end-of-life diagnosis. This study’s findings suggest that this preliminary step is at the root of an important difference: in some of these data it can be seen that the interviewer has to work hard to get participants to focus on end-of-life care for people dying of age-related general decline. This can be seen in the next excerpt, where the participant replies quite hesitantly to a reminder about the interview focus, a reminder given after a long story about a very old man dying of cancer:

‘When you say frail and elderly, I think I’m thinking of frail and elderly with cancer, but you’re, I don’t think you’re … not particularly, it’s not that ... it’s just frail elderly end of life.’

(Jen, community nurse, OOH)

In her initial choice of story and subsequent hesitation, Jen indicates tacit assumptions about the kinds of people and medical conditions that lead to an end-oflife diagnosis. Other participants reference these assumptions explicitly:

*‘Everybody looks at end of life as being* [...] *cancer-driven or long-term condition driven, I don’t see that the elderly or frail actually come into that remit.’*

(Hattie, community nurse, OOH)

*‘We wouldn’t have a kind of unexpected, a frail elderly come in* [to a ‘palliative care’ bed]. ’

(Kath, community hospital)

Some participants, however, describe ways to make the diagnostic process work to help a frail older patient, providing them with an end-of-life diagnosis:

‘You have to pigeon-hole them into whatever services would be available.’

(Gill, community nurse, OOH)

In the next excerpt, May explains how this pigeon-holing works for an old, frail patient whose health declines after admission to a ‘rehab’ bed in her unit:

*‘The doctor will find a diagnosis because at 98 you’re going to find something that’s going to kill you very soon, and they will say it’s the heart failure and then they become palliative* [...] *it’s just knowing what to put on that piece of paper to allow it to happen.’*

(May, community hospital)

May’s account helpfully disrupts the idea that making an end-of-life diagnosis is a fact-based, rule-governed process, carried on outside the complex collection of practices through which clinicians care for patients approaching the end of their lives. Instead, *‘find* [ing] *a diagnosis’* appears as an integral constituent of that web of care practices. This model of complex articulations between practices offers a better fit with this study’s data than models that portray end-of-life diagnosis leading to end-of-life care in a tidy linear relationship. By focusing guidelines and practice primarily on care needs, and relegating diagnosis to a secondary role, care might be improved for the kind of person May portrays here:

*‘I think it still hits us by surprise when it comes to the end* [...] *somebody in the community who hasn’t got a diagnosis but you know they’re reaching the end of their life* [...] *because they’re, they’re old, it’s the only reason. Well you know they’ve got a bit of this and a bit of that* [...] *we used to call it acopia, they can’t call it “not coping” anymore.’*

(May, community hospital)

Thus it is not enough simply to say that the patient needs additional care; to get this, they first need an end-of-life diagnosis. Next, the way this diagnosis affects care provision is considered.

### What effects does an end-of-life diagnosis have on care provision?

The participants portray an end-of-life diagnosis in three roles: allowing access to additional services; giving the diagnosed patient priority over other patients requesting care; and helping trigger and facilitate advance care planning. Each of these roles is more visible in some care settings than others.

#### Enabling access

The gatekeeping role of a diagnosis is visible in the accounts of participants working both inside and outside the ‘gates’. For instance, when asked what would happen if her community hospital relaxed its rule that palliative care beds are reserved for people with an end-of-life diagnosis, May replies that she would *‘be afraid to open the floodgates’*. Kath, who works at a different community hospital, does offer a suggestion to help frail older people who do not have an end-of-life diagnosis, tacitly acknowledging that they would benefit if they could get in:

*‘One solution is* [...] *that we have more units like this* [...] *you’d fill them up very quickly, that’s the only thing.’*

(Kath, community hospital)

The group of staff interviewed together at a hospice go further, dismissing the idea of broadening their admission criteria:

‘There would never be enough beds in the hospice environment to accommodate the frail elderly.’

(Group1)

Gill mirrors this statement; speaking from outside the floodgates, in the context of community OOH nursing, she describes her experience of the local hospice’s provision over several years:

*‘* [At] *first* [...] *it would only look after patients with cancer diagnoses, then it changed to any end-of-life condition, but it didn’t cover elderly, frail and I don’t know whether it does now* [...] *there isn’t a specific service for those.’*

(Gill, community nurse, OOH)

These data all indicate that older people without an end-of-life diagnosis are disadvantaged as regards access to community in-patient care close to the end of life. Another OOH community nurse spells this out:

*‘A cancer diagnosis* [...] *opens up so many doors for people, regardless of your age* [...] *if you’re frail and elderly and just dying* [...] *what have you got?* [...] *It’s a lot more closed doors.’*

(Jen, community nurse, OOH)

#### Prioritisation

A second way in which older people may be disadvantaged is surfaced by community OOH nurses’ accounts of prioritising their work. They are the only group of participants to talk about prioritisation, presumably because, unlike those working in community in-patient settings, the OOH service has no ‘ *floodgates*’ to control patient numbers. As Jen explains:

‘You never know, one night we might have one patient, one night we might have your fourteen.’

(Jen, community nurse, OOH)

Like other participants, the OOH nurses explicitly state that they provide good care, but some of the data suggest a more nuanced picture. For instance, this is Hattie’s reply when asked whether she can visit all the patients she is asked to see:

*‘I’d say 90% of the time we could* [...] *Priorities will be admission avoidance, end of life and catheter problems* [...] *but there are a lot of calls that we do* [...] *re-triage* [...] *and say, “I’m sorry, we can’t get to you”.’*

(Hattie, community nurse, OOH)

This statement, with its slightly uneasy disparity between *‘90%’* visited and *‘a lot of calls’* declined, complicates the positive framing of prioritisation visible in this next excerpt:

‘I think the beauty of out-of-hours, even though we are completely stretched beyond capacity, is that we absolutely prioritise end-of-life care.’

(Jen, community nurse, OOH)

By prioritising end-of-life care, Jen is doing exactly what healthcare guidelines recommend, but Hattie’s account shows that in resource-constrained settings this recommendation inevitably disadvantages people who are not prioritised.

#### Advance care planning

The importance of establishing the patient’s preferences regarding their end-of-life care (a process known as advance care planning) features prominently in guidance such as the Gold Standards Framework,^15^ where it is cited as a reason to make an end-of-life diagnosis. Looking at the way providers in different care settings talked about advance care planning, an interesting difference was visible: such planning is seldom mentioned in most settings, but it is foregrounded by participants working in nursing homes. For example, at the start of her interview, Iris responds to a general invitation to talk about end-of-life care by mentioning *‘start* [ing] *the discussion’* in her first sentence:

‘I think end-of-life care is very, very important, you know, we start the discussion, you know, soon after somebody come to a home.’

(Iris, nursing home)

Iris goes on to explain that the topics discussed include preferences about resuscitation and hospital admission; the new resident’s family is usually involved in these discussions, which are *‘documented and kept in our folder’*. Implicitly, all those involved accept that the resident is approaching the end of their life, and Iris refers to *‘an end-of-life care plan’* as the key product of the discussion process. Liz, who works at a different nursing home, also talks about regular meetings with every resident’s family, at which *‘part of what we do* [...] *is discuss end-of-life’*, although she and Iris both volunteer that some people are reluctant to engage with these discussions. A normative flavour is visible in this presentation of advance care planning as *‘part of what we do’* despite people’s reluctance, and in Iris’ comment that *‘some people they tend to delay the process* [of discussing end-of-life care] *’*.

Despite these hints that advance care planning is done routinely, not just offered, the nursing home managers emphasise that it is done for the patient’s benefit, helping them achieve *‘the death that they want* [...] *a nice death’*. Outside the nursing homes, in contrast, Hattie presents planning as something done primarily by and for the clinical team, with patients and relatives implicitly relegated to a box-ticking role:

*‘I believe that there should be processes, I’m not saying they’ve got to be 100% stuck to but if there’s a clear line of what happens as the patient is deemed end of life or terminal* [...] *the box has been ticked, preferred place of care, got Just in Case meds, we’ve got the next of kin* [...] *I think that’s crucial. And we haven’t got it yet.’*

(Hattie, community nurse, OOH)

Other participants do not mention advance care planning except when prompted by the interviewer. Jen’s response to such a prompt, below, is less enthusiastic than Hattie’s, perhaps because she indicates awareness of an ethical problem raised by advance planning, rather than focusing her response on efficient care provision:

*‘Er ... yes, yes, I don’t think* [advance care planning is] *going to hurt* [...] *The only thing I’m thinking of though is people never know what they’re planning for.’*

(Jen, community nurse OOH)

Although guidelines state an end-of-life diagnosis enables advance care planning, in the setting where planning featured most prominently, nursing homes, there was never a reference to residents having an end-of-life diagnosis. This paradox can be explained by a tacit assumption: moving into a nursing home constitutes a surrogate marker that makes it appropriate to begin advance care planning, an explanation supported by the next excerpt. Iris’ nursing home recently adopted the Gold Standard Framework, instead of a previous guideline, but she implies that guidelines about end-of-life care have only a limited effect on her care practices:

*‘Whatever policy comes and goes* [...] *the basic care is the same* [...] *providing holistic care,* [...] *making sure that the person is pain-free* [...] *clean, comfortable,* [...] *peaceful* [...] *that’s basic care* [...] *’*

(Iris, nursing home)

Thus good *‘basic care’* is what all residents need, and Iris’ account suggests that everyone in her nursing home receives this good care; in this setting, an end-of-life diagnosis is not needed to *‘open doors’.* In other settings, however, the study’s findings suggest that care providers privilege and prioritise patients identified as ‘end-of-life’, inadvertently disadvantaging people who die without an end-of-life diagnosis.

## DISCUSSION

### Summary

Drawing on interviews with professional providers of community end-of-life care, this study examines the role an end-of-life diagnosis plays in shaping that care. Its findings support the widely-accepted view that an end-of-life diagnosis facilitates the provision of good care: the participants say that it helps them plan and prioritise care. An end-of-life diagnosis also *‘opens doors’* to better care for the individual diagnosed, helping providers allocate additional care not available to others.

Beyond this unanimity, however, a difference was identified between providers in different settings, particularly regarding the kinds of patients who are likely to obtain or not obtain an end-of-life diagnosis. Providers in NHS-funded settings identify end-of-life diagnosis as problematic in frail older people who have no single life-limiting condition. This difficulty about end-of-life diagnosis is sometimes identified as a barrier to good community care; some participants describe circumventing this barrier by *‘pigeon-holing’* older people into an acceptable end-of-life diagnostic category. In contrast, concerns about diagnosis and prognosis are invisible in the accounts of participants working in private nursing homes. These accounts implicitly present admission to a nursing home as a marker indicating that it would be unsurprising if the resident was to die in the foreseeable future. This assumption helps staff offer good end-of-life care to their residents, at least in these particular nursing homes.

A further salient finding came only from the interviews with community nurses, who provide a reactive service: to meet unpredictable demands, they have to prioritise, seeing end-of-life patients quickly while ‘re-triaging’ others. Inevitably, prioritisation risks disadvantaging those others, including frail older people who die without an end-of-life diagnosis; this is an unintended consequence of policies and practices centred on end-of-life diagnosis.

### Strengths and limitations

By focusing on providers’ accounts and using the sociology of diagnosis to understand them, this study was able to highlight how end-of-life diagnosis shapes care provision. Recruiting participants from several different care settings made it possible to use comparisons both within the data and with the broader literature.

As well as constituting a strength, the wide variety of different settings also potentially limits the validity of the comparisons. This issue is compounded by the differing definitions of ‘end-of-life’ used by participants working in different settings, making it hard to generalise about what constitutes adequate end-of-life care; care needs in the last days of life may well differ from care needs over the last months.

Further limitations arose from the small number of interviews and the use of snowball sampling: providers liable to offer more negative accounts of their practice may have felt disinclined or simply too busy to volunteer. Recruiting within a small area and interviewing in the workplace may have raised concerns about anonymity.

### Comparison with existing literature

It has long been recognised that, compared to younger people, older people are less likely to access satisfactory end-of-life care at home or in a hospice, and are more likely to die in hospital.^22^^–^^24^ In a study of hospital admissions in the last days of life,^25^ both family and professional carers described these admissions as necessitated by the impossibility of accessing adequate support and palliative care in community settings. This study’s findings underline the challenge of providing such support and care. To address this challenge, participants working in resource-constrained community settings prioritise patients who have an end-of-life diagnosis, or use the diagnosis to obtain extra resources, practices that inevitably disadvantage frail older people who are less likely to be diagnosed as ‘end-of-life’ or ‘for palliative care’.^13^^,^^14^ This unintended consequence is particularly problematic as frail older people constitute a growing proportion of those who die each year, prompting calls for *‘a shift in thinking towards reframing ... end-of-life care to meet the growing needs of the ageing population’*.^26^

A growing body of research and policy responds to this call primarily by enjoining practitioners to make more end-of-life diagnoses, particularly in frail older people; frailty has been established as a life-limiting condition^27^ meriting palliative care, although the multiplicity of different definitions and scoring systems complicate research and practice.^28^ As the findings of this study show, end-of-life diagnosis illustrates the power of a diagnosis to *‘determin*[e] *who has access to what resources’*,^29^ particularly salient where resources are constrained. Like some of the participants in this study, GPs in other studies raise concerns about the workload implications of increasing their palliative care register by including *‘frail or elderly patients’*.^13^^,^^23^ In addition to resource constraints, these studies identify a concern implicitly related to the identity conferred by an end-of-life diagnosis.^29^ Harrison *et al*,^14^ for example, describes GPs’ reluctance to *‘talk to patients too early’*. Such *‘talk’* requires the practitioner to offer an end-of-life diagnosis; many frail older people may be unready to identify themselves as candidates for palliative care. The authors found that practitioners, too, may not readily identify such people as candidates for end-of-life diagnosis; this finding echoes Pocock *et al* ’s report^23^ that their GP participants:

*‘struggled to put patients with nonmalignant diagnoses on the* [palliative care] *register* [...] *as* [...] *a consequence of there not being the same intellectual link between these patients and the need for EOL* [end-of-life] *care’.*

Thomas and Gray^26^ attempt to address these concerns, offering a positively-framed account in which the value of an end-of-life diagnosis is explicitly linked to its ability to *‘enable a more proactive, less crisis-led approach’* to care. Crucially, however, they also suggest that commissioners *‘might consider increasing investment in community services’*, including district nurses and night sitters. The district nurse participants of this study described themselves as *‘stretched beyond capacity ’*, strongly supporting the case for this increased investment.

The findings of this study highlight an inequity of service provision that has been described and discussed elsewhere,^30^ sometimes specifically related to dementia^31^ or cast more broadly as ageism.^8^ Covinsky *et al* ^19^ relate this inequity to the central role of end-of-life diagnosis, concluding that:

*‘end-of-life care systems that are targeted toward patients with functional trajectories clearly suggesting impending death* [...] *are poorly suited to older people dying with progressive frailty’*.

Over a decade later, Lloyd *et al* ^32^ state that: *‘the current palliative care model is problematic for* [frail older people] *’*.

The current authors suggest that, to solve its problems, this model should begin by reconsidering its reliance on establishing an end-of-life diagnosis.

### Implications for practice

These findings suggest that, as policymakers intend, an end-of-life diagnosis benefits its recipient. However, they also highlight an unintended effect of policies centred on end-of-life diagnosis: such policies risk privileging people with a single life-limiting condition over people who die of a collection of age-related conditions. To mitigate this risk requires a rethink: is the ‘end-of-life’ lens useful for thinking about how best to support frail older people and their families? More specifically, is it helpful to try to distinguish between a crisis that turns out to be at the end of life, and an identical crisis that turns out to be a temporary and reversible episode?

Given the uncertainty of prognosis in this group of people, it is suggested that practitioners and policymakers should avoid increasing their focus on end-oflife diagnosis, and instead focus primarily on what each frail older individual needs. This shift would inform a more nuanced approach to clinicians’ conversations about care priorities, enabling them to situate such conversations within the web of practices through which they provide care to older people. For policymakers, commissioners, and managers, it would help to clarify the challenge of providing good end-of-life care equitably to all groups within their populations.
